# Genetic Predisposition of Anti-Cytomegalovirus Immunoglobulin G Levels and the Risk of 9 Cardiovascular Diseases

**DOI:** 10.3389/fcimb.2022.884298

**Published:** 2022-06-27

**Authors:** Jiang-Shan Tan, Jia-Meng Ren, Luyun Fan, Yuhao Wei, Song Hu, Sheng-Song Zhu, Yanmin Yang, Jun Cai

**Affiliations:** ^1^ Emergency Center, Fuwai Hospital, National Center for Cardiovascular Diseases, National Clinical Research Center of Cardiovascular Diseases, Chinese Academy of Medical Sciences and Peking Union Medical College, Beijing, China; ^2^ Department of Cardiology and Institute of Vascular Medicine, Peking University Third Hospital, NHC Key Laboratory of Cardiovascular Molecular Biology and Regulatory Peptides, Key Laboratory of Molecular Cardiovascular Science, Ministry of Education. Beijing Key Laboratory of Cardiovascular Receptors Research, Beijing, China; ^3^ Hypertension Center, FuWai Hospital, State Key Laboratory of Cardiovascular Disease, National Center for Cardiovascular Diseases, Peking Union Medical College, Chinese Academy of Medical Sciences, Beijing, China; ^4^ Peking University China-Japan Friendship School of Clinical Medicine, Beijing, China; ^5^ Center for Respiratory and Pulmonary Vascular Diseases, Department of Cardiology, Key Laboratory of Pulmonary Vascular Medicine, National Clinical Research Center of Cardiovascular Diseases, State Key Laboratory of Cardiovascular Disease, Fuwai Hospital, National Center for Cardiovascular Diseases, Chinese Academy of Medical Sciences and Peking Union Medical College, Beijing, China

**Keywords:** cytomegalovirus, cardiovascular disease, Mendelian randomization, immunology, risk

## Abstract

**Background:**

Accumulating evidence has indicated that persistent human cytomegalovirus (HCMV) infection is associated with several cardiovascular diseases including atherosclerosis and coronary artery disease. However, whether there is a causal association between the level of anti-HCMV immune response and the risk of cardiovascular diseases remains unknown.

**Methods:**

Single-nucleotide polymorphisms associated with anti-cytomegalovirus immunoglobulin (Ig) G levels were used as instrumental variables to estimate the causal effect of anti-cytomegalovirus IgG levels on 9 cardiovascular diseases (including atrial fibrillation, coronary artery disease, hypertension, heart failure, peripheral artery disease, pulmonary embolism, deep vein thrombosis of the lower extremities, rheumatic valve diseases, and non-rheumatic valve diseases). For each cardiovascular disease, Mendelian randomization (MR) analyses were performed. Inverse variance-weighted meta-analysis (IVW) with a random-effects model was used as a principal analysis. In addition to this, the weighted median approach and MR-Egger method were used for further sensitivity analysis.

**Results:**

In the IVW analysis, genetically predicted anti-cytomegalovirus IgG levels were suggestively associated with coronary artery disease with an odds ratio (OR) of 1.076 [95% CI, 1.009–1.147; p = 0.025], peripheral artery disease (OR 1.709; 95% CI, 1.039–2.812; p = 0.035), and deep vein thrombosis (OR 1.002; 95% CI, 1.000–1.004; p = 0.025). In the further analysis, similar causal associations were obtained from weighted median analysis and MR-Egger analysis with lower precision. No notable heterogeneities and horizontal pleiotropies were observed (p > 0.05).

**Conclusions/Interpretation:**

Our findings first provide direct evidence that genetic predisposition of anti-cytomegalovirus IgG levels increases the risk of coronary artery disease, peripheral artery disease, and deep vein thrombosis.

## Introduction

Cardiovascular diseases (CVDs) are the leading cause contributing to mortality and morbidity worldwide. In China, national economic losses caused by CVDs are estimated to approach $8.8 trillion between 2012 and 2030 (Bloom, 2013). Although multifactorial contributors such as genetic factors, environmental factors, and immune and inflammatory factors are identified and under exploration in the pathogenesis of CVDs, studies have increased in recent indicating the role of microbiome (Finlay and Humans, 2020) in non-communicable diseases (NCDs) including CVDs, with the Human Microbiome Project initiated over 2007 and 2016. Recent studies suggest that the human microbiome, including oral and gut, contributes to the pathogenesis of inflammation (Alam et al., 2017; Bruce-Keller et al., 2018) and immune dysfunction (Kong et al., 2019), which may contribute to CVD pathophysiology.

In addition to the bacterial microbiome and their metabolites already analyzed as a pathogenic factor, biomarker, and potential intervention targets in NCDs, viruses (Zou et al., 2016) (virome) constituting a portion of the microbiome present with limited investigations. Virus infections are long-term issues for associations with CVDs (Kawana, 1985; Pothineni et al., 2017; Du et al., 2018) *via* multiple epidemiological, serological, molecular, and animal studies. Of note, the high cardiovascular manifestations under the COVID-19 pandemic (Chung et al., 2021) further underscore the importance of more explorations of viruses on CVDs. A recent study has also revealed that circulating cell-free DNA of microbiome in CVDs are enriched with bacteriophages and eukaryotic viruses (Dinakaran et al., 2014) in circulation compared with healthy controls. Moreover, viral metagenomic profiling of fecal samples in patients with coronary heart disease revealed reduced gut virome of the family *Microviridae (* Guo et al., 2017 *)*, a viral taxon dominant in the healthy population, but the direct correlation between virome and coronary heart disease remains unclear. The above evidence indicates that viruses (virome) are involved in the development of CVDs.

Cytomegalovirus (CMV) is one of the double-stranded DNA (dsDNA) viruses that belong to the herpes virus family with relatively common prevalence and is under investigation for association with CVDs. Many observational studies have revealed the association between CMV and CVDs. A meta-analysis of community-based prospective studies covering 34,564 subjects and 4,789 patients with CVDs including coronary artery disease, ischemic heart disease, stroke, and heart failure reveals a 22% increase in CVD (Wang et al., 2017) incidence risk after CMV infection. Seropositivity of CMV, particularly CMV immunoglobulin (Ig) G levels (Roberts et al., 2010), are also observed as predictors of all-cause and/or CVD-related mortality in elderly Swedes (Wikby et al., 2005), Latinos (Hadrup et al., 2006), and Americans with higher C-reactive protein (CRP) levels (Simanek et al., 2011). In addition, although not in total accordance, histopathological studies (Zhu and Liu, 2020) detected CMV DNA in serum of coronary artery disease patients (Westphal et al., 2006) and atherosclerotic plaque or vascular wall specimens (Shi and Tokunaga, 2002; Gredmark-Russ et al., 2009). A rare propensity of vascular thrombosis after CMV infections has also been observed since the 1980s (Boers and Haak, 1984). The potential contribution of CMV to multiple CVDs may thus be indicated. However, it is worth noting that we cannot provide evidence for a causal association between CMV and CVDs. First, all the aforementioned findings are based on observational studies. Therefore, no conclusion can yet be reached about whether individuals infected with CMV would be at higher risk of CVDs or vice versa. Moreover, findings based on observational studies may have been affected by some confounders, even some unknown or unmeasured risk factors.

Mendelian randomization (MR) is a novel computational method to assess the causal associations between risk factors and particular diseases (Smith and Ebrahim, 2003; Davey Smith and Hemani, 2014; Davies et al., 2018) due to the following reasons: 1) the allocation of genetic variants is entirely random; 2) the inherited genetic variants have been determined at conception and will not change due to non-differential measurement error or confounding. In this study, we first investigate the role of CMVs in all available CVDs in the databases, including atrial fibrillation, coronary artery disease, hypertension, heart failure, peripheral artery disease, pulmonary embolism (PE), deep vein thrombosis (DVT) of the lower extremities, rheumatic valve diseases, and non-rheumatic valve diseases, by using 2-sample summary MR based on the genetic variants of anti-cytomegalovirus IgG levels as instruments.

## Material and Methods

### Overall Study Design

**Figure 1:**
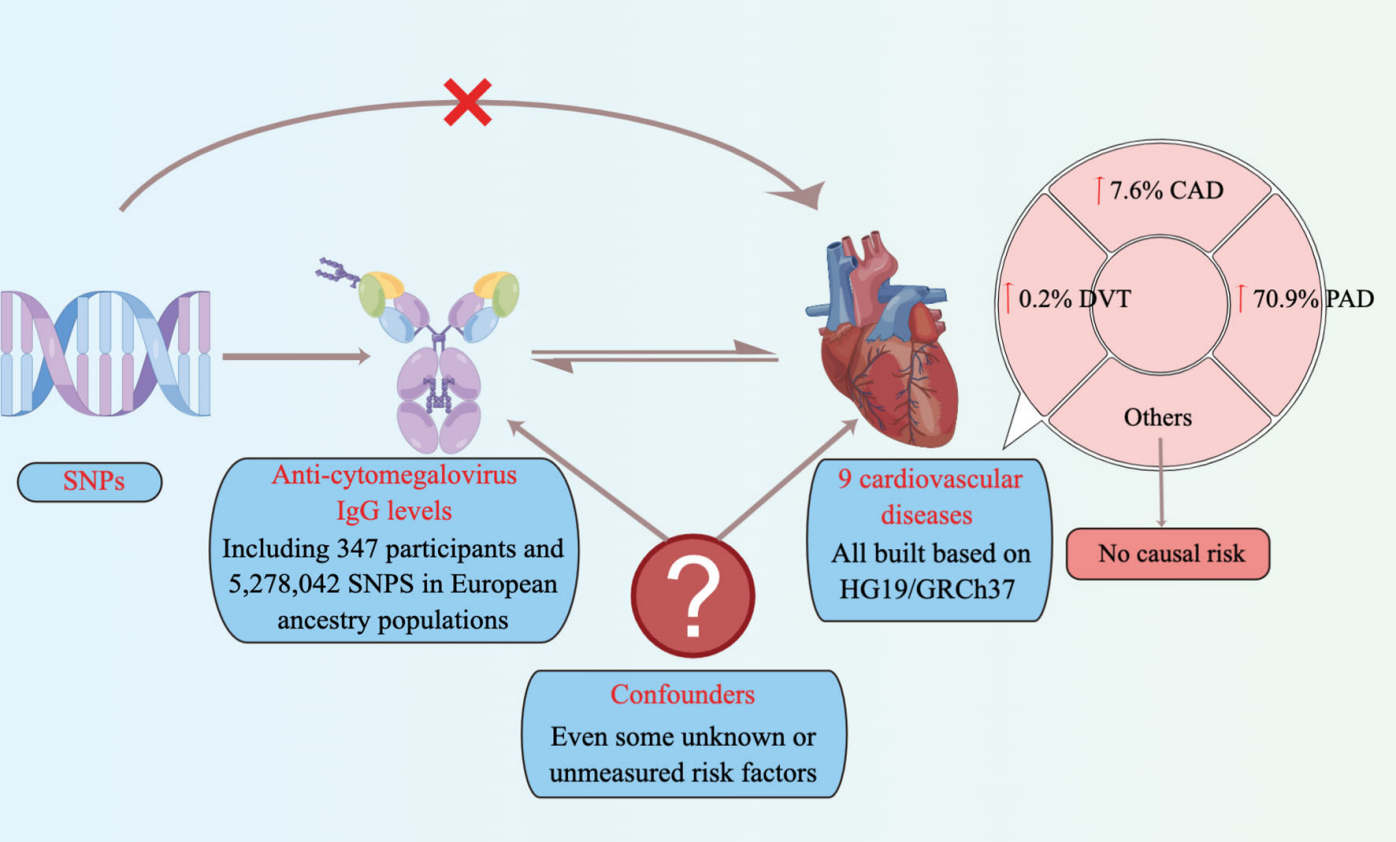
Schematic representation of an MR analysis. We selected SNPs associated with anti-cytomegalovirus IgG levels, and the corresponding effect for these SNPs was estimated based on the risk of 9 cardiovascular diseases. Because of the randomization and independence of alleles at meiosis, MR is a powerfully predictive tool to assess causal relationships with no bias inherent to observational study designs. CAD, coronary artery disease; PAD, Peripheral artery disease; DVT, deep vein thrombosis; MR, Mendelian randomization; SNPs, single-nucleotide polymorphisms.

In the present study, the summary data were used for a two-sample MR (Richmond et al., 2016; Lawlor, 2016) analysis to assess the causal association between anti-cytomegalovirus IgG levels and the risk of 9 CVDs (**Figure 1**). Due to the fact that the summary data were obtained from previously published and the ethics approvals have been obtained in their institutions, no additional ethics approvals were required in this study.

### Data Sources

#### Exposure: Anti-Cytomegalovirus IgG Levels

In the present MR study, single-nucleotide polymorphisms (SNPs) of the exposure are used as inverse variances (IVs). Summary statistics of anti-cytomegalovirus IgG levels were obtained from completed genome-wide association study (GWAS) summary data on protein levels as described by Sun et al. in 2018 (Sun et al., 2018). The basic information on anti-cytomegalovirus IgG levels (including 347 participants and 5,278,042 SNPs) is shown in **Table 1**.

**Table 1:** Characteristics of GWASs in anti-cytomegalovirus IgG levels and the risk of 9 cardiovascular diseases.

Exposure/outcomes	No. of controls	No. of cases	Sample size	Year of publication	Number of SNPs	Build	Study population
Anti-cytomegalovirus IgG levels (Scepanovic et al., 2018)	–	–	347	2018	5,278,042	HG19/GRCh37	European
Atrial fibrillation (Christophersen et al., 2017)	102,776	15,979	118,755	2017	10,719,646	HG19/GRCh37	European
Coronary artery disease (van der Harst and Verweij, 2018)	424,528	122,733	547,261	2017	7,934,254	HG19/GRCh37	European
Hypertension (2018)	359,957	1,237	361,194	2018	9,646,741	HG19/GRCh37	European
Heart failure (2021)	195,091	13,087	208,178	2021	16,380,422	HG19/GRCh37	European
Peripheral artery disease (2020)	92,349	2,383	94,732	2020	16,152,119	HG19/GRCh37	European
Pulmonary embolism (2022)	95,023	1,366	96,389	2020	16,152,119	HG19/GRCh37	European
DVT of lower extremities (2020)	359,078	2,116	361,194	2018	10,544,982	HG19/GRCh37	European
Rheumatic valve diseases (2020)	96,273	162	96,435	2020	16,152,119	HG19/GRCh37	European
Non-rheumatic valve diseases (2018)	359,588	1,606	361,194	2018	10,080,950	HG19/GRCh37	European

DVT, deep vein thrombosis; GWASs, genome-wide association studies; SNPs, single-nucleotide polymorphisms.

Genetic variants were selected as instruments if they satisfied the uncorrelated (r^2^ < 0.001) SNPs, which were significant risk factors based on a threshold of the genome-wide level of statistical significance (p < 5 × 10^−7^). Moreover*, SNPs are independent of each other to avoid offsets caused by linkage disequilibrium (LD), and the LD of SNPs associated with anti-cytomegalovirus IgG must meet the* r^2^ < 0*.001, window size = 10,000 kb. The 1000 Genomes Project (*1000 Genomes Project Consortium, 2010*) based on European samples was used to estimate the LD levels. Moreover, the F statistic of SNPs was used to screen the SNPs with a high correlation between instrumental variables and exposure factors. It is generally considered to exclude the bias of weak instrumental variables when the F statistic >10. F statistics = (β/SE)^2^.*

In addition to those mentioned above, three other assumptions were implemented. First, the IVs must be associated with anti-cytomegalovirus IgG levels (IV assumption 1). Second, the IVs affect the risk of CVDs only *via* anti-cytomegalovirus IgG levels (IV assumption 2). Third, the measured or unmeasured confounders were not involved in the IVs (IV assumption 3) (Hemani et al., 2018).

### Study Outcome: Cardiovascular Diseases

Corresponding data for CVDs were obtained from completed GWAS summary data in the MR platform, which were available at https://gwas.mrcieu.ac.uk/. To assess the causal association between anti-cytomegalovirus IgG levels and differential cardiovascular outcomes, a broad range of CVDs were analyzed, including atrial fibrillation, coronary artery disease, hypertension, heart failure, peripheral artery disease, PE, DVT of the lower extremities, rheumatic valve diseases, and non-rheumatic valve diseases. If more than one previously published GWAS is available, the newest and largest one with detailed publication information is preferred in the present analysis. The detailed information on CVDs involved in this study is shown in **Table 1**.

### Statistical Analysis

Due to no individual-level GWAS data being available, the two-sample MR analysis was used to assess the causal effect of anti-cytomegalovirus IgG levels on CVDs (**Figure 1**), which has been described previously (Burgess et al., 2013).

In the present MR analysis, an IV-weighted meta-analysis (IVW) with a random-effects model was used as a principal analysis (Larsson et al., 2020). In addition to this, the weighted median approach (Burgess et al., 2017) and MR-Egger method (Bowden et al., 2015) were used for further sensitivity analysis to reduce the bias due to horizontal pleiotropy. Comparing the consistency of three different methods, which were based on different horizontal pleiotropy, can help to judge the reliability of the causal association between anti-cytomegalovirus IgG levels and CVDs (Burgess et al., 2015; Xu et al., 2017). In addition, MR Pleiotropy RESidual Sum and Outlier (MR-PRESSO) was used to remove SNPs with pleiotropic outliers (p < 0.1). Two-tailed p < 0.05 was used in all statistical tests. Bonferroni-corrected analysis was used with a threshold of p < 0.006 (a *=* 0.05/9 outcomes) (Liu et al., 2021). Associations with p-values between 0.006 and 0.05 were considered suggestive evidence of causal associations, requiring further confirmation (Liu et al., 2021). All statistical analyses were finished with the R version 4.0.3, TwoSampleMR version 0.5.5, and MRPRESSO version 1.0 (Verbanck et al., 2018; Broadbent et al., 2020).

## Results

### Genetic Instrumental Variables for Anti-Cytomegalovirus IgG Levels

The essential information regarding the enrolled GWAS studies is shown in **Table 1**. In total, 10 GWAS studies (including 1 GWAS of anti-cytomegalovirus IgG levels and 9 GWAS of CVDs) and 2,126,770 individuals were included in the present study. Moreover, 4 independent variants for anti-cytomegalovirus IgG levels (**Table 2**) were selected as instrumental SNPs based on a GWAS significance of p < 5 × 10^−7^ and an *LD* threshold of r^2^ < 0.001. With reference to other MR studies, a relaxed statistical threshold for genetic instruments was used once a few significant SNPs are available (Gage et al., 2017; Choi et al., 2019).

**Table 2:** List of genetic instruments for anti-cytomegalovirus igg levels by each instrumental SNP (GWAS significance with p < 5 × 10^−7^ and linkage disequilibrium threshold with R^2^ < 0.001).

No.	SNP	Locus	Chr.	EA	OA	EAF	β (SE)
1	rs7583185	–	2	G	A	0.0597668	−0.218 (0.042)
2	rs1001036	–	8	T	G	0.0553936	−0.256 (0.045)
3	rs1600519	–	11	A	C	0.110787	−0.167 (0.029)
4	rs35701456	LOC101927605	16	C	A	0.0364431	−0.272 (0.050)

Note. Chr., chromosome; EA, effect allele; OA, other alleles; EAF, effect allele frequency; SNP, single-nucleotide polymorphism; GWAS, genome-wide association study.

### Effects of Anti-Cytomegalovirus IgG Levels on 9 Cardiovascular Diseases

**Figure 2:**
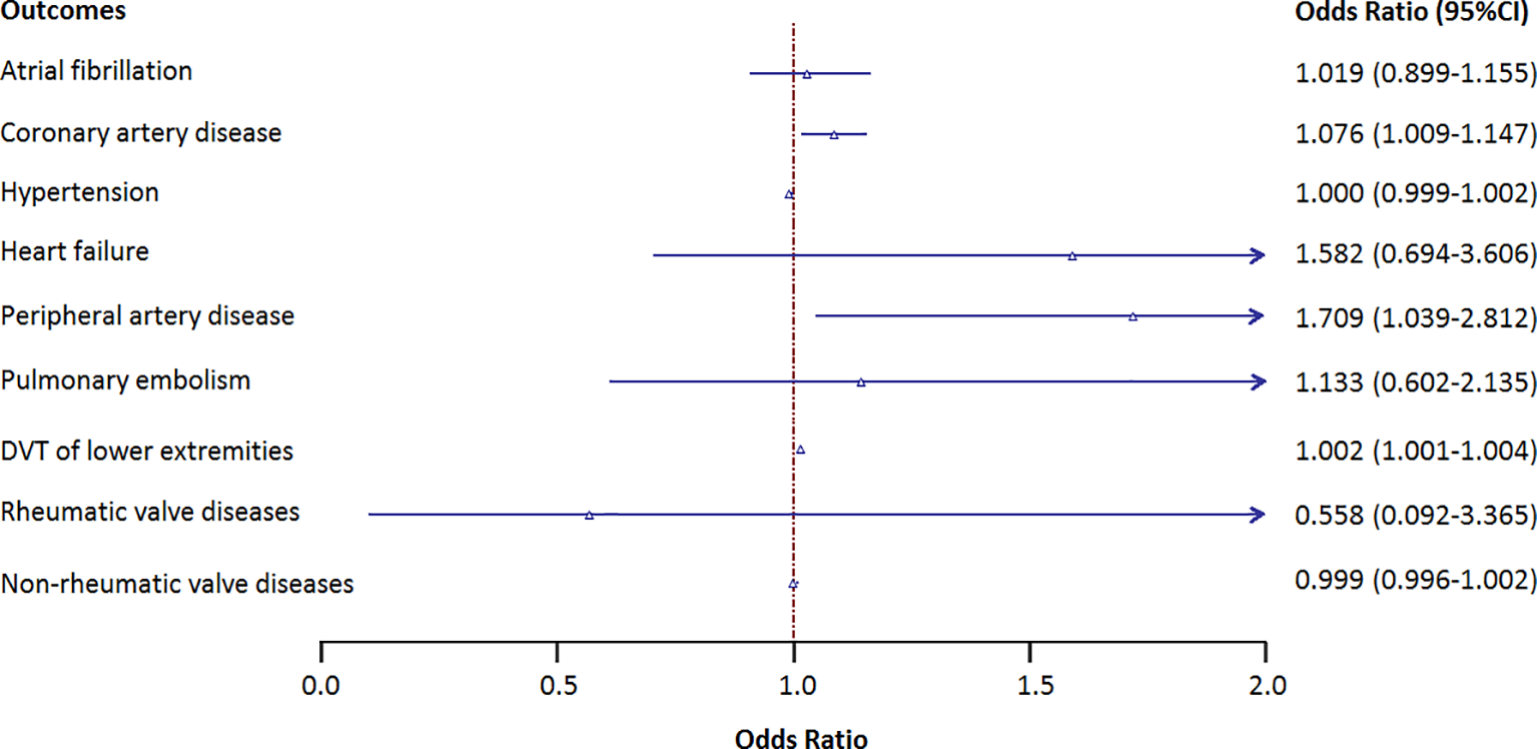
Results of the Mendelian randomization analysis investigating the association of genetically proxied anti-cytomegalovirus IgG levels and risk of 9 cardiovascular diseases. Forest plot showing inverse variance-weighted Mendelian randomization estimates for the association between anti-cytomegalovirus IgG levels and risk of 9 cardiovascular diseases. DVT, deep vein thrombosis; OR, odds ratio.

**Figure 2** shows the causal association between genetically proxied anti-cytomegalovirus IgG levels and 9 CVDs. There is a suggestive causal association between genetically predicted anti-cytomegalovirus IgG levels and an increased risk of coronary artery disease with an odds ratio (OR) of 1.076 [95% CI, 1.009–1.147; p = 0.025], peripheral artery disease with an OR of 1.709 (95% CI, 1.039–2.812; p = 0.035), and DVT with an OR of 1.002 (95% CI, 1.000–1.004; p = 0.025). No causal association was observed between anti-cytomegalovirus IgG levels and other CVDs.

### Sensitivity Analysis for Mendelian Randomization Analysis

To guarantee the reliability of our causal association, the OR estimates of the weighted median analysis and MR-Egger analysis were used as sensitivity analysis. Similar suggestive associations were obtained from weighted median analysis and MR-Egger analysis with lower precision (**Table 3**).

**Table 3:** Associations between genetically predicted anti-cytomegalovirus IgG levels and 9 CVDs in sensitivity analyses using the weighted median and MR-Egger methods.

	Weighted median		MR-Egger	
Outcome	OR (95% CI)	p-Value	OR (95% CI)	p-Value
Atrial fibrillation	1.012 (0.872–1.174)	0.877	1.273 (0.643–2.521)	0.560
Coronary artery disease	1.076 (0.996–1.163)	0.063	0.941 (0.676–1.311)	0.755
Hypertension	1.000 (0.999–1.002)	0.760	1.001 (0.994–1.007)	0.827
DVT of lower extremities	1.002 (1.000–1.004)	0.052	1.003 (0.995–1.012)	0.539
Non-rheumatic valve diseases	0.999 (0.997–1.001)	0.472	0.997 (0.981–1.013)	0.753

Note. DVT, deep vein thrombosis; OR, odds ratio; MR, Mendelian randomization.

### Analysis of Heterogeneity and Horizontal Pleiotropy

No notable heterogeneity was observed in the modified Cochran Q statistics across SNPs of different CVDs (p > 0.05). Moreover, no horizontal pleiotropy was observed in the MR pleiotropic tests (intercept p-value >0.05) and MR-PRESSO.

## Discussion

Herein, we explored the causal association between genetically predicted anti-cytomegalovirus IgG levels and the risk of 9 CVDs. In the present MR, we first found direct evidence that genetically predicted higher anti-cytomegalovirus IgG levels were causally associated with a higher risk of coronary artery disease, peripheral artery disease, and DVT.

The human microbiome includes the oral and gut microbiome. Many studies have revealed that both gut and oral microbiome may be modified by systemic diseases, including CVDs (Graves et al., 2019). The collection of microbes living in the human intestinal tract and oral cavity has been known to influence more than digestion. Indeed, the microbiota, which collectively is widely considered the body’s largest endocrine organ, can generate biologically active metabolites and impact many aspects of host physiology (Witkowski et al., 2020). Human CMV is a prevalent beta-herpes virus with approximately 50% European and American population under infection (Bate et al., 2010) and is difficult to be cleared from the host. With such common prevalence and higher cardiovascular complications after CMV infection in observational studies, whether the observational association is causal remains unclear. CMV infection enables a lifetime latent phase with capacities of infecting cells, generally epithelial cells and peripheral blood mononuclear cells, and reactivation with higher titers of IgG. Interestingly, CMV also affects vessel wall cells, with its infection linked to coronary heart disease and atherosclerosis (Wang et al., 2017; Zhu and Liu, 2020), which was also consistent with the conclusions of our study, revealing a causal association between genetic predisposition of anti-cytomegalovirus IgG levels and higher risk of coronary artery disease. Notably, our study first establishes the causative correlation for CMV IgG level in peripheral artery disease, a disease that mainly shares similar mechanisms of atherosclerosis with coronary artery disease but involves peripheral vessel beds, in line with a population-based case–control study (Bloemenkamp et al., 2002) previously observing a positive correlation in women between CMV IgG titer and peripheral artery disease (OR = 1.6, 95% CI, 1.1–2.3). The presence of CMV antigen and DNA is observed in both human cardiovascular samples and animal models (Melnick et al., 1983; Melnick et al., 1994). CMV as an intracellular pathogen could involve diverse phases of atherogenesis (Pothineni et al., 2017) including endothelium activation, leucocyte migration, lipid core formation, smooth muscle proliferation, plaque stability, and thrombus formation (Strååt et al., 2009). Other systemic mechanisms of inflammation and host immune response interplaying with CMV (Forte et al., 2020), and molecular mimicry between serum antibodies of CMV and host proteins such as heat shock protein 60 (Mayr et al., 2000), are also in a recent investigation. The CMV and its IgG antibody as biomarkers for atherosclerotic diseases such as peripheral artery disease and coronary artery disease, and their pathogenic roles need further exploration. Additionally, CMV is a novel target for CVD prevention and treatment *via* therapies such as antiviral drugs, chimeric antigen receptor T cells, immunotherapy, or CMV vaccines (Vasilieva et al., 2020) (i.e., Triplex, a Modified Vaccinia Ankara (MVA) vaccine encoding CMV antigens (La Rosa et al., 2017; Aldoss et al., 2020), Poxvirus Vectored Cytomegalovirus Vaccine) may be developed.

Moreover, we first confirmed the causal association between genetic predisposition of anti-CMV IgG level and DVT, a thrombotic disorder of the veins with a higher risk for life-threatening PE. Although with the limited investigation, several case reports or case series have revealed a trend for correlation between CMV infection and venous thrombosis-related diseases such as venous thromboembolism (VTE; the combination of DVT and PE) (Ceccarelli et al., 2018), portal vein thrombosis (Squizzato et al., 2007), and superior mesenteric vein thrombosis (Walter et al., 2021). Additionally, case–control studies observed a higher incidence rate of thrombosis in the CMV infection group (6.4% vs. 0%) (Atzmony et al., 2010) and a higher rate of anti-CMV IgG positivity in the VTE group compared with healthy controls (Schimanski et al., 2012). A meta-analysis analyzed 97 reports of thrombosis associated with CMV infection, presenting DVT and PE as the most prevalent diseases, particularly in immunocompromised populations (Justo et al., 2011). A recent retrospective study analyzed 1,007 VTE patients and observed 0.1% co-occurrence with acute CMV infection, predominantly in women and the younger population (Yildiz et al., 2016). In addition, a prospective study covering approximately 90,000 VTE patients demonstrated anti-CMV IgM seropositivity as an over 2-fold independent risk factor for VTE onset (Paran et al., 2013). Plausible mechanisms for thrombosis after CMV infection include the following: 1) infection of endothelial cells promoting their procoagulant activity, such as Virchow’s triad activation (Sherman et al., 2014); 2) transient hypercoagulable state after anti-phospholipid antibody formation caused by CMV-derived peptide, which mimics human beta-2 glycoprotein 1 (Uthman and Gharavi, 2002; Gharavi et al., 2002; Nakayama et al., 2014); 3) host response to phospholipids from CMV envelopes with procoagulant propensities, for instance, phosphatidylserine (Pryzdial and Wright, 1994); 4) platelet aggregation *via* upregulated production of von Willebrand factor and expression of ICAM-1 and VCAM-1 (Rahbar and Söderberg-Nauclér, 2005). Interestingly, the host procoagulant reaction to CMV infection appears to be transient with a window period after infection and thrombosis (Justo et al., 2011). Anticoagulant and antiviral therapy choices and duration in those populations may be further explored with close monitoring for virus and host statuses.

In the present MR, we have implemented some key measures to satisfy the assumptions of MR analysis. First, only statistically significant SNPs of anti-cytomegalovirus IgG levels based on a genome-wide significant level valid association were used in our MR analysis to satisfy the IV assumption 1 as described in our methods. To minimize the bias and confounders in anti-cytomegalovirus IgG levels and CVDs, we only chose the GWAS of anti-cytomegalovirus IgG levels and CVDs, which were finished just in European ancestry populations. Therefore, the potential bias and confounders in the present study are small. To ensure that these SNPs only affect CVDs through anti-cytomegalovirus IgG levels (no pleiotropic effects), MR-Egger regression was performed, and no evidence of directional pleiotropic effects was observed in our MR.

Despite these strengths, some limitations are still worth noting. First, anti-cytomegalovirus IgG levels can only represent a state after infection, and it is not sufficient for us to assess the viral infection activity or acute infection. Second, even though the effect size of anti-cytomegalovirus IgG levels is quite modest, it is estimated that a large number of patients are at an increased risk of coronary artery disease, peripheral artery disease, and DVT because of the large population with anti-cytomegalovirus IgG. Most importantly, it has been widely accepted that the risk factors are various in populations with different races and ethnicities (Tan et al., 2021). However, all of the summary-level statistics in the present MR were based on European ancestry populations. Therefore, further studies are needed to clarify whether the conclusions apply to other populations.

In essence, our MR analysis first revealed the casual association between genetic predisposition of anti-cytomegalovirus IgG levels and the risk of coronary artery disease, peripheral artery disease, and DVT. Future studies on CMV infection in the pathogenesis of atherosclerosis and thrombosis, as well as anti-CMV therapy development for disease prevention, are of significance. In addition, preventive anticoagulation treatment may be investigated for individuals infected by CMV.

## Data Availability Statement

The datasets presented in this study can be found in online repositories. The names of the repository/repositories and accession number(s) can be found in the article/supplementary material.

## Ethics Statement

Ethical approval was not provided for this study on human participants because the summary data were previously published and the ethics approvals have been obtained in their institutions. Therefore, no additional ethics approvals were required in this study. The patients/participants provided their written informed consent to participate in this study.

## Author Contributions

JC contributed to the conception and design of the study. J-ST organized the database. J-ST and LF performed the statistical analysis and wrote the first draft of the manuscript. YY contributed to interpret the results and review the revised manuscript. J-MR and S-SZ helped with the revision of the manuscript. All authors contributed to manuscript revision and read and approved the submitted version.

## Funding

The work was supported by grants from Capital‘s Funds for Research and Application of Clinical Diagnosis and Technology(Z191100006619121), Treatment National Center for Clinical Research in Cardiovascular Diseases (No. NCRC2020015), the CAMS Innovation Fund for Medical (CIFMS, Sciences 2021-I2M-1-007 and 2022-GSP-GG-26), Natural National Science Foundation of China (Project ID. 81825002), Beijing Outstanding Young Scientist Program (Project ID. BJJWZYJH01201910023029), and the project for the distinguishing academic discipline of Fuwai hospital (Project ID. 2022-FWQN02).

## Conflict of Interest

The authors declare that the research was conducted in the absence of any commercial or financial relationships that could be construed as a potential conflict of interest.

## Publisher’s Note

All claims expressed in this article are solely those of the authors and do not necessarily represent those of their affiliated organizations, or those of the publisher, the editors and the reviewers. Any product that may be evaluated in this article, or claim that may be made by its manufacturer, is not guaranteed or endorsed by the publisher.
